# Digit-tracking reveals curiosity-driven visual attention in macaque monkeys

**DOI:** 10.1038/s41598-026-57654-4

**Published:** 2026-06-14

**Authors:** Yidong Yang, Antoine Ameloot, Guillaume Lio, Angela Sirigu, Jean-René Duhamel

**Affiliations:** 1 Institut des Sciences Cognitives Marc Jeannerod, CNRS, UMR 5229, Université Claude Bernard Lyon, 67 Bd Pinel, 69675 Bron Cedex, France; 2 IMind Center of Excellence for Autism, Le Vinatier Hospital, Bron, France; 3 Institute of Neuroscience la Timone, UMR7289, CNRS, Aix-Marseille University, Marseille, France

**Keywords:** Neuroscience, Psychology, Psychology

## Abstract

We used digit-tracking, a touch-based method for assessing visual attention, to investigate spontaneous exploration in macaque monkeys. By engaging with degraded images on a touch-sensitive display, monkeys could uncover high-resolution portions through finger movements, allowing for natural and unrestricted interaction. Monkeys received juice rewards after touching a predetermined number of pixels, but no specific regions were targeted. Attention maps were generated from their interactions, along with data from human digit-tracking and monkey eye-tracking experiments. Direct comparisons across recording methods revealed that monkey digit-tracking attention maps were significantly correlated with both monkey eye-tracking and human digit-tracking maps, indicating shared patterns of visual exploration across species and devices. We further applied a saliency model and a Convolutional Neural Network (CNN) model to predict the empirical explorations. The correlation between model prediction maps and empirical attention maps indicated that monkeys focused non-randomly on information-rich regions, with the CNN model providing the most accurate predictions. These findings suggest that exploration was driven by intrinsic curiosity, beyond the extrinsic rewards for interaction. Digit-tracking offers a minimally invasive, portable alternative to eye-tracking, expanding research opportunities in visual cognition within ecologically valid settings.

**Supplementary Information:**

The online version contains supplementary material available at https://doi.org/10.1038/s41598-026-57654-4.

## Introduction

Studies in non-human primates have significantly advanced our understanding of the mechanisms underlying visual attention in humans, largely due to the remarkable similarities in the organization of visual perception and cognition across species. These similarities enable researchers to draw insights from primate behavior to inform our understanding of human visual processing, including how both monkeys and humans explore visual images and prioritize relevant information. In both species, exploration tends to focus on visually salient regions^1,2^, such as the relevant features of objects^3^, body parts, and faces, which are key to survival and social interaction^4–6^. The preference for faces can be modulated by factors such as sex, rank and social perception features of the face^7–10^. Understanding how primates attend to and process visual stimuli thus offers valuable perspectives on the neural and cognitive mechanisms that govern attention in humans.

Traditionally, studies investigating visual attention in non-human primates have relied on eye-tracking methods. Although eye-tracking provides precise measures of visual exploration, these methods are often constrained by the need for extensive training^11–13^. Additionally, its use in nonhuman primates typically requires the surgical implantation of a head restraining device to stabilize the head during calibration and recording, which constitutes an invasive procedure and limits the flexibility and portability of such studies.

Digit-tracking, a method wherein subjects explore images by using their fingers on a touch-sensitive display, presents a promising alternative to traditional eye-tracking^14^. By allowing for spontaneous and natural exploration without the need for head restraint or eye movement calibration, digit-tracking eliminates many of the constraints associated with eye-tracking studies. In this method, a Gaussian-blurred image is presented on a touch-sensitive interface and can be locally unblurred by sliding a finger over it. The blur simulates the low resolution of the peripheral retina, while the clear area revealed by touching the screen models the high foveal acuity, and eye movements are inferred from finger movements. This method has already been validated in human subjects, where it has proven to be an effective and minimally invasive technique for studying visual attention^14–16^. However, its application in non-human primates has yet to be thoroughly explored.

In this study, we aimed to test the feasibility and validity of digit-tracking in a group of laboratory macaques. We hypothesized that monkeys engage in visual exploration driven by intrinsic curiosity and predicted that they would demonstrate non-random, spontaneous exploration patterns by focusing on visually informative regions of the display, despite the absence of explicit cues or instructions to do so. We further hypothesized that digit-tracking exploration patterns would correspond to those obtained with established measures of visual attention, and therefore directly correlate with attention maps derived from monkey eye-tracking and human digit-tracking. Finally, we hypothesized that a Convolutional Neural Network (CNN) model could predict these exploration patterns based on image features.

## Results

**Fig. 1:**
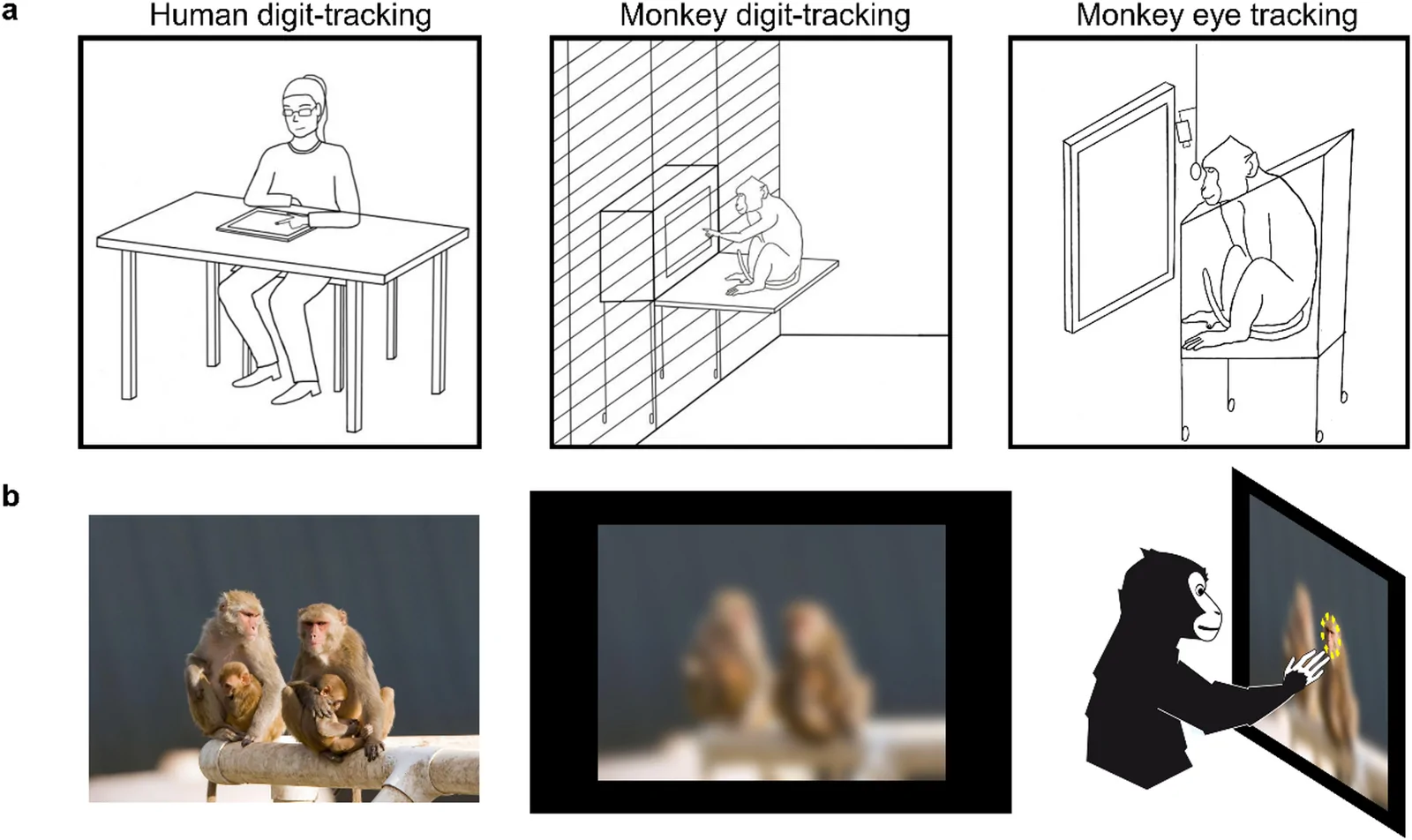
Overview of the experimental setup and digit-tracking paradigm. (**a**) Schematic illustration of the three experimental conditions used in this study: human digit-tracking, monkey digit-tracking, and monkey eye-tracking. In human digit-tracking, the participant explored the stimuli on a tablet. In monkey digit-tracking, the monkey interacts with the tablet integrated into a mobile device which is positioned outside the cage. In monkey eye-tracking, the monkey is seated in a primate chair with head restrained. (**b**) Illustration of stimulus presentation in the digit-tracking task, showing an example of the original image, the Gaussian-blurred image, and the blurred image with a clear aperture revealed by finger contact. The blurred image is displayed within a black screen background, and the clear aperture reveals a local high-resolution region slightly offset from the point of contact to prevent occlusion by the finger.

We trained five macaques to freely view 59 images – featuring animals, humans, objects and landscapes – using digit-tracking within their home cage. For validation, we tested nine human subjects with the digit-tracking method and two of the macaques with standard eye-tracking, all using the same images (Fig. 1). We began by evaluating the training procedure through an analysis of each monkey’s performance and generated attention maps to visualize their exploration patterns. We then directly compared attention maps across recording methods to assess the correspondence between monkey digit-tracking, monkey eye-tracking, and human digit-tracking. Finally, we used a standard saliency model and a Convolutional Neural Network model to predict the empirical attention maps from monkey digit-tracking, human digit-tracking, and monkey eye-tracking.

### Monkeys were engaged in the digit-tracking task

**Fig. 2:**
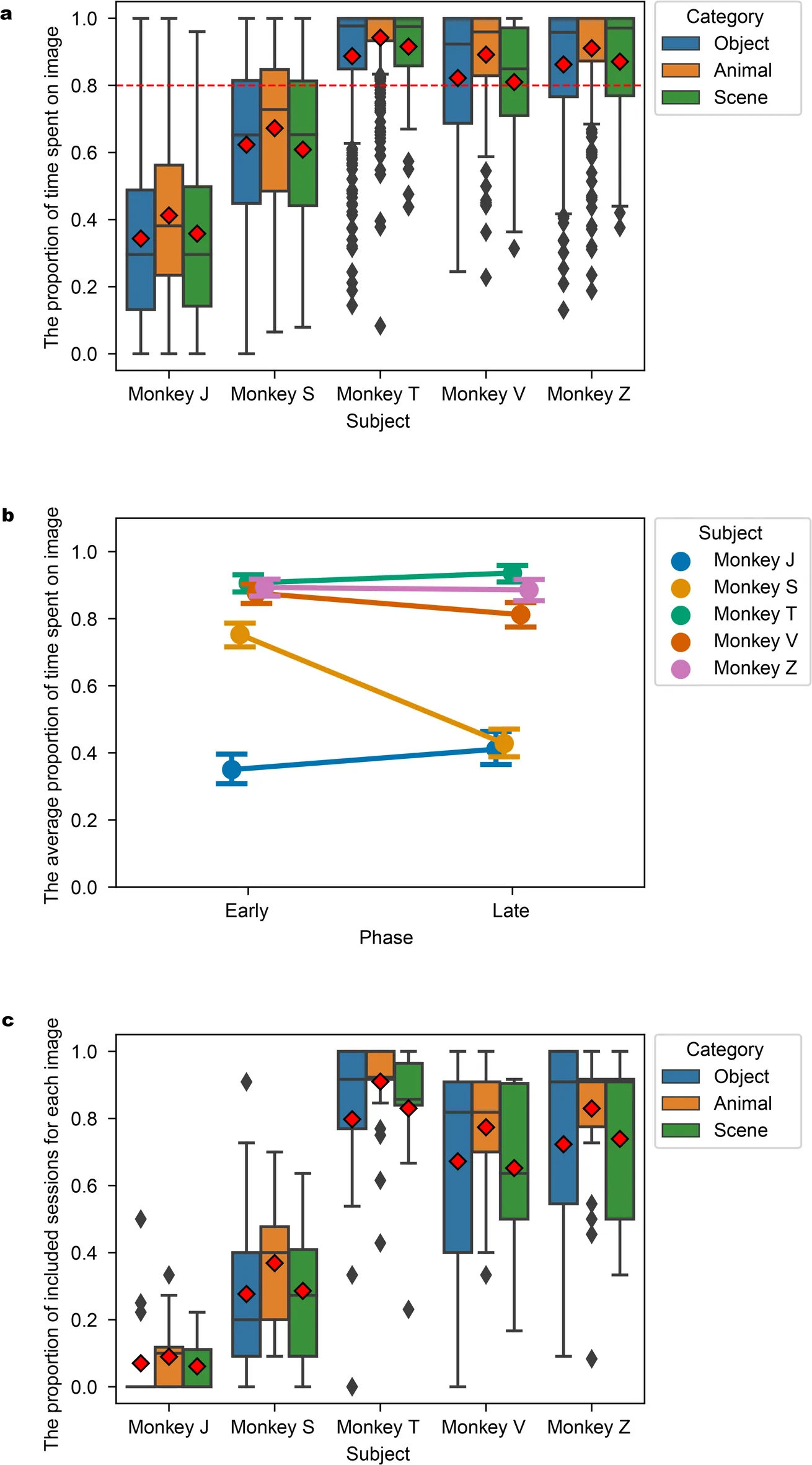
The inclusion of data based on the proportion of time spent on images. (**a**) Boxplots showing the proportion of time spent on images (as opposed to the background) of each trial grouped by subject and category. Diamonds indicate mean values. Monkey S and Monkey J exhibited a large proportion of trials in which they spent considerable time on the background rather than the image. The horizontal red dashed line at 0.8 denotes the inclusion threshold used to select trials for subsequent analyses. (**b**) Changes in the mean proportion of time spent on images between early and late phases of the experiment. “Early” corresponds to the first third of recorded sessions and “late” to the final third. Each point represents the subject-level mean proportion of exploration time within image, averaged across all images and sessions within the corresponding phase (early or late), and error bars indicate the standard error of the mean (SE). Post hoc comparisons revealed significant decreases in on-image time for Monkeys V and S, whereas Monkey J showed an increase. The pronounced change in Monkey S may be related to the longer interval between its early and late phase. (**c**) Boxplots showing the proportion of sessions retained for analysis after filtering based on the on-image time criterion. For each subject and image category, the proportion was computed separately for each image as the number of sessions in which that image met the inclusion criterion divided by the total number of sessions for that subject. Boxes summarize the distribution of these image-level proportions across images within each category. Only a small number of sessions from Monkeys J and S remained after filtering.

Monkeys were trained to explore images on a touchscreen integrated into a mobile, battery-powered device equipped with a liquid delivery system. The device was positioned directly against their home cages, allowing them to perform the task in a low-constraint, familiar environment. Monkeys were not incentivized to touch specific areas. They could touch any location on the screen and a reward was automatically delivered after a predetermined number of pixels had been touched. The stimulus set comprised images spanning a wide range of content. To capture theoretically relevant differences in how visual content guides attention, images were grouped into three categories (Fig. S1): object, scene, and animal (including humans). This categorization reflects well-established distinctions between stimuli primarily driven by low-level visual features (objects and scenes) and those carrying high ethological and social significance (animals), which are known to engage attentional and exploratory mechanisms differently. During the familiarization and training procedures, the cumulative exploration distance required to complete a trial—measured as the distance traveled by the monkey’s finger across the screen—was progressively increased to a final criterion of approximately 56° of visual angle. On average, it took 9 training sessions for the monkeys to reach this final criterion and complete the entire image set within a single session, with individual variations ranging from 7 to 11 sessions. To assess whether the monkeys were genuinely engaged with the images, we calculated the proportion of their exploration time spent within the image boundaries. Although this measure cannot establish genuine preference for specific images, it can at least exclude trials in which the animals were almost certainly not engaging meaningfully with the visual content. Because the images did not occupy the entire surface of the touchscreen, trials in which the monkeys’ fingers landed predominantly outside the image region suggested that they had adopted a strategy to obtain rewards without attending to the image itself. Figure 2a shows that three of the monkeys mainly focused on the image contents, especially images of animals.

To examine whether engagement with the image content increased over the course of the experiment, sessions were ordered chronologically for each subject, and the first and last third of sessions (rounded down to the nearest integer) were defined as early and late phase, respectively. The mean values of the proportion of exploration time spent within the image boundaries were entered into a two-way ANOVA with Subject and Phase (early vs. late) as factors. We observed a significant interaction, *F*(4,290) = 91.436, *p* < 0.001, $\:{\eta\:}_{p}^{2}$ = 0.558. Specifically, post hoc results indicated that Monkey V (t(290) = 3.957, $\:{p}_{bonf}$ = 0.004, 95% CI [0.011, 0.117], d = 0.451) and Monkey S (t(290) = 20.091, $\:{p}_{bonf}$ < 0.001, 95% CI [0.272, 0.378], Cohen’s d = 2.290) showed a significant decrease over sessions, whereas Monkey J (t(290) = −3.762, $\:{p}_{bonf}$ = 0.009, 95% CI [−0.114, −0.008], Cohen’s d = 0.429) showed a significant improvement (Fig. 2b). We observed that only Monkey S showed a large change, whereas the other subjects either did not show significant differences or exhibited changes smaller than 0.12. Notably, the early and late phase for Monkey S were separated by several months, whereas the other monkeys completed their sessions within a more condensed timeframe. This extended interval may account for the pronounced difference observed in Monkey S. Overall, however, the subjects did not exhibit a dramatic reduction of engagement in image exploration during the late phase.

Subsequent analyses were performed on the attention maps derived from finger trajectories (see Figure S2 for examples of finger trajectories) which showed the allocation of attention during exploration, so we deemed it reasonable to exclude trials where the subjects did not exhibit sufficient task engagement, defined as touches within the image boundaries. We set the inclusion criteria for the proportion of time spent on the image at 0.8. Then, we computed the proportion of included sessions for each image. Figure 2c shows the proportion of included sessions for each image and subject. We found that only a few sessions from Monkey J and Monkey S met the inclusion criteria, which were insufficient for further analysis. Therefore, we excluded these two subjects. After excluding certain images and subjects, the average number of included sessions for the remaining subjects was as follows: Monkey T had an average of 11.138 sessions (SD = 2.267), Monkey V had an average of 8.121 sessions (SD = 2.760), and Monkey Z had an average of 8.678 sessions (SD = 2.956).

### Properties and reliability of attention maps

**Fig. 3:**
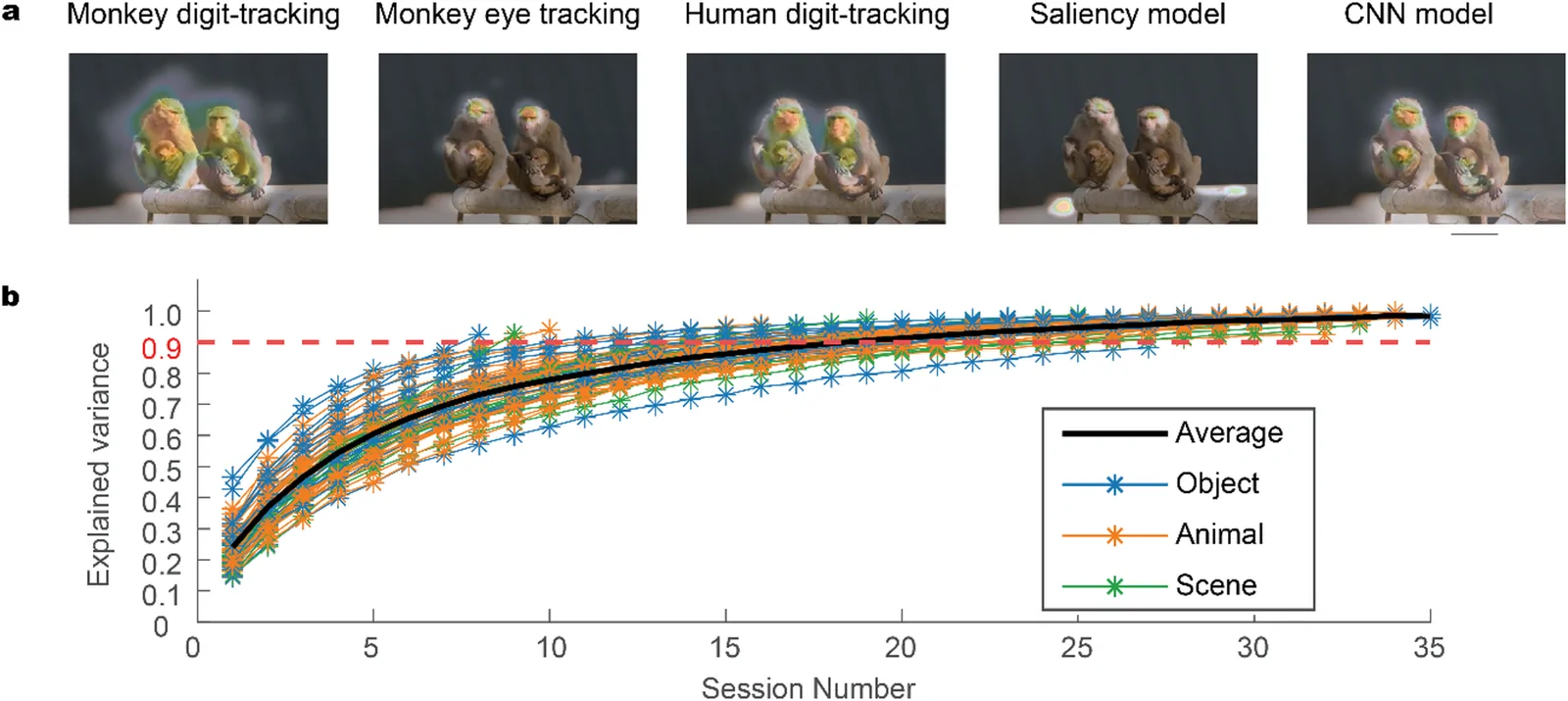
Properties and reliability of attention maps. (**a**) Comparison of attention maps for the same image collected across different experiments, shown alongside prediction maps generated by two computational models. Warmer colors indicate regions of higher exploration. High-density regions (“hot spots”) are primarily located on objects, bodies, and faces, consistent with salient visual features. (**b**) Relationship between explained variance and the number of sessions included in the analysis. The curves show the cumulative variance explained as more sessions are added. On average, approximately 18 sessions were required to account for 90% of the variance, indicating the amount of data needed to obtain stable attention maps in this paradigm.

To present the exploration patterns, we generated empirical attention maps for each trial and computed the average attention maps across subjects and sessions. Figure 3a shows the average attention maps for one image from different data sources. Visual inspection of the attention maps suggested that explorations were often directed towards objects, contrasts, features, and socially relevant elements. In particular, for images containing living animals, the maps showed more exploration in facial regions, in line with patterns reported in previous eye-tracking studies^4–6^. The exploration patterns showed similarities across different data sources, which we will quantify in the next section.

To determine the number of sessions needed for reliable measurements, we performed a convergence analysis as follows: For each image, we (1) generated average attention maps using incrementally larger session samples (from *N* = 1 to the maximum available), (2) calculated the correlation between these sample-based maps and the reference map (derived from all available sessions), and (3) plotted the mean correlation coefficients against session count (Fig. 3b). The analysis revealed that an average of 17.69 sessions (SD = 4.09) were required to achieve 90% of the maximum explainable variance. This estimate was robust across our dataset, with ≥ 18 sessions available for analysis from at least 3 subjects on 51 different images. For comparison, Lio et al.^14^ reported that only about 7 human participants were needed to account for 95% of the variance. While the human and monkey cases are not directly comparable—the former reflecting variability across participants and the latter across sessions within individuals—both analyses illustrate how repeated sampling helps stabilize exploratory maps. Importantly, our convergence analysis not only characterizes the reliability of the present dataset, but also provides a practical benchmark for future studies by indicating the approximate number of sessions likely needed to obtain stable and reliable measurements.

### Shared attention patterns across species and methods

To quantify the similarities suggested by the attention maps, we examined how attention maps derived from monkey digit-tracking relate to those obtained using monkey eye-tracking and human digit-tracking. For each recording method, we first computed session-averaged maps for each subject and each image. These subject-level maps were then averaged across subjects, yielding one mean attention map per image for each recording method. We then computed correlations between the monkey digit-tracking attention maps and the corresponding maps obtained from monkey eye-tracking and human digit-tracking. This analysis allowed us to evaluate whether digit-tracking captures attention patterns that are consistent across species and across recording methods.

**Fig. 4:**
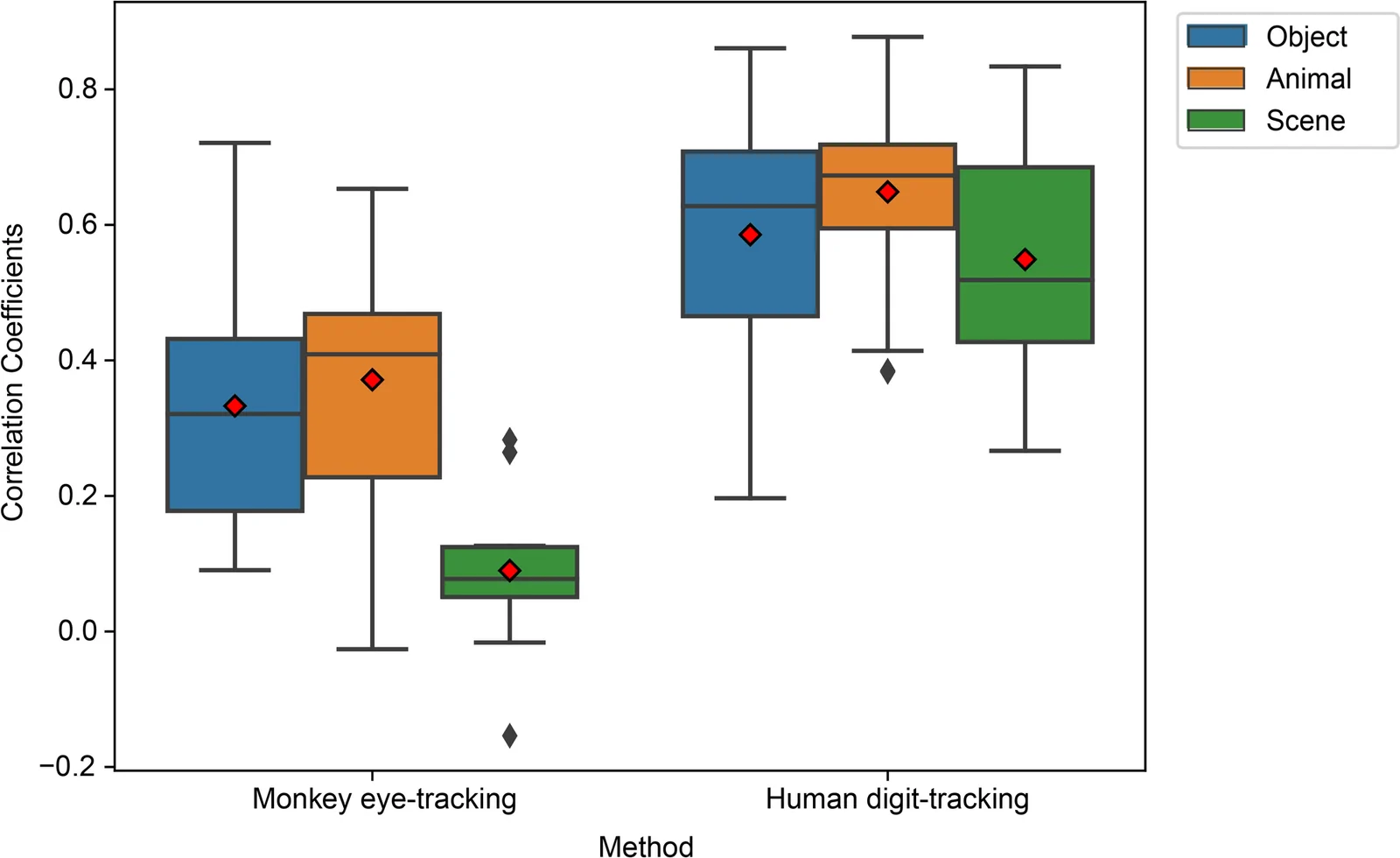
Correlations between attention maps from monkey digit-tracking and other two recording methods. Boxplots show correlation coefficients between attention maps derived from monkey digit-tracking and those obtained from monkey eye-tracking (left) and human digit-tracking (right). Colors indicate image categories (object, animal, scene). Red diamonds denote mean values. Mean correlation coefficients (± SD) between attention maps derived from monkey digit-tracking and other two recording methods are shown respectively (Monkey eye tracking: object, 0.332 ± 0.178; animal, 0.371 ± 0.174; scene, 0.090 ± 0.121; Human digit-tracking: object, 0.586 ± 0.188; animal, 0.649 ± 0.130; scene, 0.549 ± 0.169). Correlations with human digit-tracking are consistently higher than those with monkey eye-tracking across categories. Category effects differ across methods: correlations with human digit-tracking do not differ significantly across categories, whereas correlations with monkey eye-tracking are higher for animal and object images than for scene images.

We ran an ANOVA with Method and Category as factors on the correlation coefficients (see Fig. 4). Because correlation coefficients are not normally distributed, values were Fisher-transformed prior to ANOVA. The analysis revealed significant main effects of Method (*F*(1,56) = 131.621, *p* < 0.001, $\:{\eta\:}_{p}^{2}$ = 0.702) and Category (*F*(2,56) = 5.860, *p* = 0.005, $\:{\eta\:}_{p}^{2}$ = 0.173), with no significant interaction (*F*(2,56) = 2.050, *p* = 0.138, $\:{\eta\:}_{p}^{2}$ = 0.068). Correlations with human digit-tracking were higher than correlations with monkey eye-tracking across all image categories (Object: t(56) = 5.954, $\:{p}_{bonf}$ < 0.001, 95% CI [0.173, 0.541], d = 1.515; Animal: t(56) = 7.616, $\:{p}_{bonf}$ < 0.001, 95% CI [0.241, 0.565], d = 1.709; Scene: t(56) = 6.773, $\:{p}_{bonf}$ < 0.001, 95% CI [0.307, 0.815], d = 2.381). Follow-up comparisons indicated that, for correlations with human digit-tracking, there were no significant differences among categories (all three pairwise comparisons not significant). In contrast, for correlations with monkey eye-tracking, correlations were higher for animal (t(56) = 4.416, $\:{p}_{bonf}$ < 0.001, 95% CI [0.096, 0.531], d = 1.331) and object (t(56) = 3.665, $\:{p}_{bonf}$ = 0.008, 95% CI [0.044, 0.497], d = 1.149) images than for scene images, with no difference between animal and object categories (t(56) = 0.741, $\:{p}_{bonf}$ = 1.000, 95% CI [-0.134, 0.220], d = 0.182).

Overall, monkey digit-tracking attention maps showed significant correlations with both monkey eye-tracking and human digit-tracking maps (all the correlations are significant), indicating that digit-tracking captures meaningful aspects of visual attention. The stronger correspondence observed with human digit-tracking likely reflects greater similarity in recording methods and apparatus rather than a fundamental difference in attentional processes. This effect may also be influenced by the limited number of subjects available in the eye-tracking dataset. While the robust correlations between human and monkey digit-tracking (ranging from 0.55 to 0.65) demonstrate a high degree of cross-species consistency in touch-based exploration, the relatively moderate alignment between monkey digit-tracking and eye-tracking (particularly in scene images, see Fig. 4) warrants closer examination. To further contextualize the magnitude of these correlations, we assessed within-subject reliability by computing inter-session correlations in eye tracking data. For each subject and image, attention maps from different sessions were pairwise correlated and averaged, yielding mean inter-session correlations of Monkey T: 0.306 (SD = 0.256, animal), 0.339 (SD = 0.269, object), 0.204 (SD = 0.221, scene) and Monkey V: 0.384 (SD = 0.237, animal), 0.403 (SD = 0.261, object), 0.230 (SD = 0.173, scene). This moderate level of within-subject consistency indicates that even for the same subject viewing the same image, attention maps recorded by the same device vary substantially across sessions. These values place a practical upper bound on the magnitude of cross-method correlations involving eye-tracking, even in the absence of systematic method differences. Consequently, neither monkey eye-tracking nor human digit-tracking can be considered a definitive “golden standard” for monkey digit-tracking. Instead, these results highlight the need to consider both shared and method-specific components when interpreting similarities and differences across these three measures of visual exploration.

### Digit-tracking captures features predicted by models

Because neither monkey eye-tracking nor human digit-tracking can be treated as a definitive reference for monkey digit-tracking, due to limited reliability and sample size, we next evaluated attentional correspondence using computational models that provide a shared feature-based reference across datasets. Specifically, we assessed how well attention maps derived from monkey digit-tracking, human digit-tracking, and monkey eye-tracking aligned with model-generated prediction maps. This indirect approach allowed us to test whether digit-tracking captures similar attentional biases as eye-tracking and whether these biases are driven by shared visual features across species. While this approach does not quantify species- or modality-specific differences directly, it provides a unified framework to evaluate attentional commonalities across datasets.

**Fig. 5:**
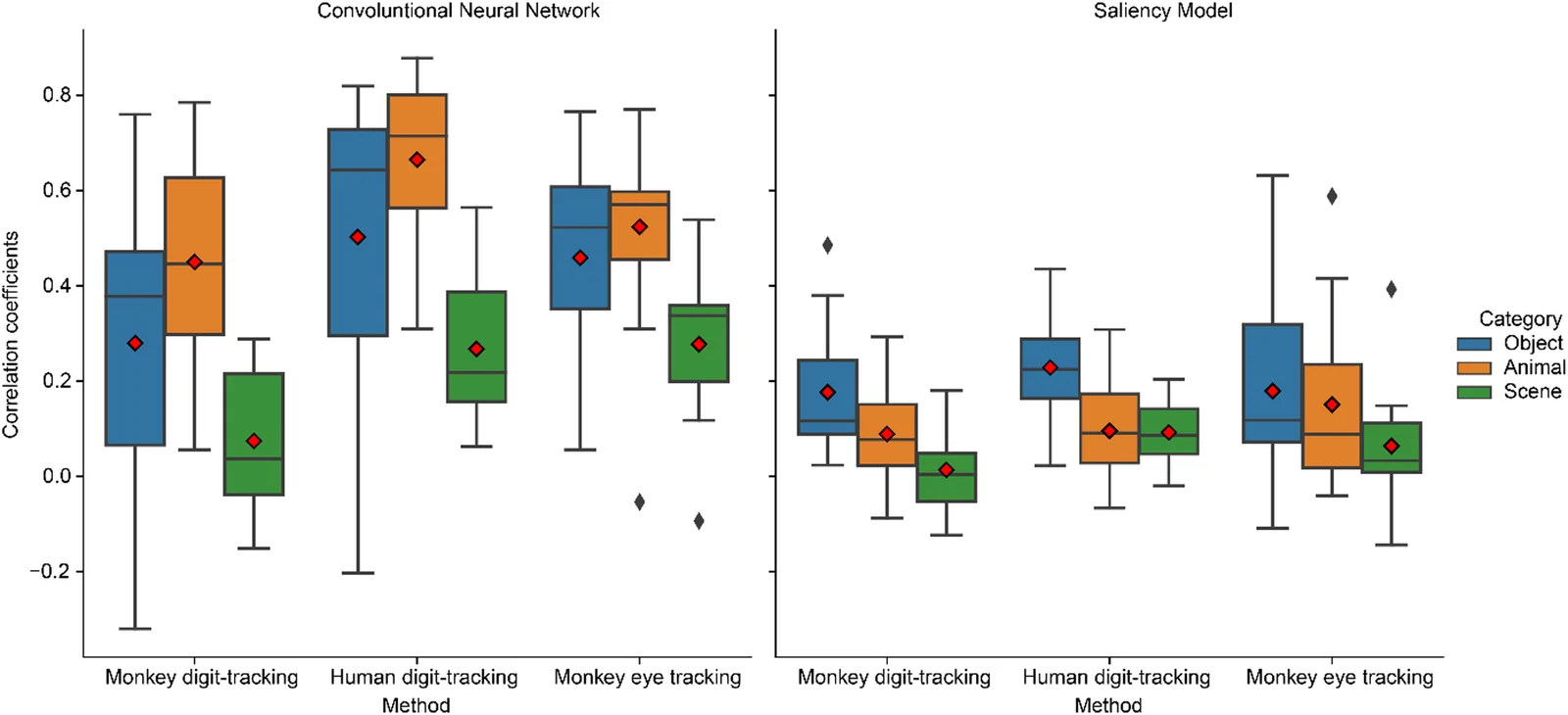
Comparisons between model prediction maps and empirical attention maps. Boxplots of correlation coefficients between model prediction maps and empirical attention maps are shown for each method and image category; diamonds indicate mean values. Correlations were computed at the image level by comparing model prediction maps with empirical attention maps averaged across subjects, yielding 59 correlation coefficients per method and model. Mean correlation coefficients (± SD) between model prediction maps and empirical attention maps are shown for the CNN and saliency models (CNN model: monkey digit-tracking—object, 0.318 ± 0.346; animal, 0.521 ± 0.290; scene, 0.076 ± 0.149; human digit-tracking—object, 0.626 ± 0.408; animal, 0.864 ± 0.314; scene, 0.286 ± 0.207; monkey eye-tracking—object, 0.523 ± 0.257; animal, 0.600 ± 0.199; scene, 0.292 ± 0.183. Saliency model: monkey digit-tracking—object, 0.182 ± 0.136; animal, 0.090 ± 0.105; scene, 0.014 ± 0.096; human digit-tracking—object, 0.236 ± 0.116; animal, 0.097 ± 0.101; scene, 0.093 ± 0.073; monkey eye-tracking—object, 0.190 ± 0.203; animal, 0.159 ± 0.185; scene, 0.066 ± 0.146). Predictions from the Convolutional Neural Network (CNN) model aligned more closely with the empirical attention maps than those from the saliency model (ANOVA: *F*(1,58) = 113.690, *p* < 0.001, $\:{\eta\:}_{p}^{2}$ = 0.662). Model performance also varied by image category: the CNN model performed best for images of animals whereas the saliency model performed relatively better for images of objects. For individual-level performance of monkey digit-tracking across categories and models, please see Fig. S3.

We selected two models with distinct strengths: (1) a saliency model^17^, which computes low-level visual features to predict bottom-up attention, (2) a Convolutional Neural Network (CNN) model (Lio et al.^14^), trained on human digit-tracking and eye-tracking data, which incorporates both low-level saliency and higher-level social cues (see Methods for details of the two models). For each method, empirical attention maps were first averaged across sessions and subjects for each image, yielding one map per image. These image-level empirical maps were then correlated with the corresponding model prediction maps, resulting in 59 correlation coefficients per method and model. Because correlation coefficients are not normally distributed, values were Fisher-transformed prior to statistical analysis. The transformed coefficients were submitted to a two-way ANOVA with Model and Method as factors. The analysis revealed a significant main effect of Model (*F*(1,58) = 113.690, *p* < 0.001, $\:{\eta\:}_{p}^{2}$ = 0.662), indicating that the CNN predictions aligned more closely with the empirical attention maps than did the saliency model (Fig. 5). This result is consistent with the presence of social content in our stimuli, which the saliency model could not capture.

To evaluate model performance across methods and stimulus categories, we conducted separate two-way ANOVAs (Method × Category) for each model. For the CNN model (see Fig. 5 left panel), significant main effects were observed for both method (*F*(2,112) = 35.699, *p* < 0.001, $\:{\eta\:}_{p}^{2}$ = 0.389) and category (*F*(2,112) = 12.534, *p* < 0.001, $\:{\eta\:}_{p}^{2}$ = 0.309), with a significant interaction (*F*(4,112) = 2.940, *p* = 0.024, $\:{\eta\:}_{p}^{2}$ = 0.095). Post hoc comparisons were conducted within each factor level to clarify the source of the interaction. Across methods, animal images consistently yielded higher correlations than scene images for all three methods (human digit-tracking: t(56) = 4.804, $\:{p}_{bonf}$ < 0.001, 95% CI [0.173, 0.982], d = 2.014; monkey digit-tracking: t(56) = 4.247, $\:{p}_{bonf}$ = 0.003, 95% CI [0.092, 0.797], d = 1.551; monkey eye-tracking: t(56) = 3.927, $\:{p}_{bonf}$ = 0.009, 95% CI [0.044, 0.571], d = 1.072). In contrast, no significant differences were observed between animal and object images, nor between object and scene images, within any method (all *p*s > 0.05). Across categories, human digit-tracking showed significantly higher correlations than monkey digit-tracking for animal (t(56) = 7.567, $\:{p}_{bonf}$ < 0.001, 95% CI [0.190, 0.495], d = 1.195) and object (t(56) = 6.001, $\:{p}_{bonf}$ < 0.001, 95% CI [0.135, 0.481], d = 1.075) images, whereas no difference was observed for scene images (*p* > 0.05). No other pairwise differences between methods were significant within any category (all *p*s > 0.05).

The saliency model yielded different results, with weaker but still significant main effects for method (*F*(2,112) = 3.498, *p* = 0.034, $\:{\eta\:}_{p}^{2}$ = 0.059) and category (*F*(2,112) = 6.685, *p* = 0.002, $\:{\eta\:}_{p}^{2}$ = 0.193), and no significant interaction (*F*(4,112) = 2.080, *p* = 0.088, $\:{\eta\:}_{p}^{2}$ = 0.069). Unlike the CNN model (see Fig. 5 right panel), the saliency model showed no significant difference between monkey digit-tracking and eye-tracking (t(56) = 1.974, $\:{p}_{bonf}$ = 0.160, 95% CI [-0.011, 0.096], d = 0.307), though human digit-tracking predictions remained more accurate than those from monkey digit-tracking (t(56) = 2.777, $\:{p}_{bonf}$ = 0.022, 95% CI [0.005, 0.088], d = 0.334). For stimulus category, the saliency model performed best for object images, outperforming both animals (t(56) = 2.644, $\:{p}_{bonf}$ = 0.032, 95% CI [0.006, 0.169], d = 0.627) and scenes (t(56) = 3.431, $\:{p}_{bonf}$ = 0.003, 95% CI [0.041, 0.249], d = 1.040).

**Fig. 6:**
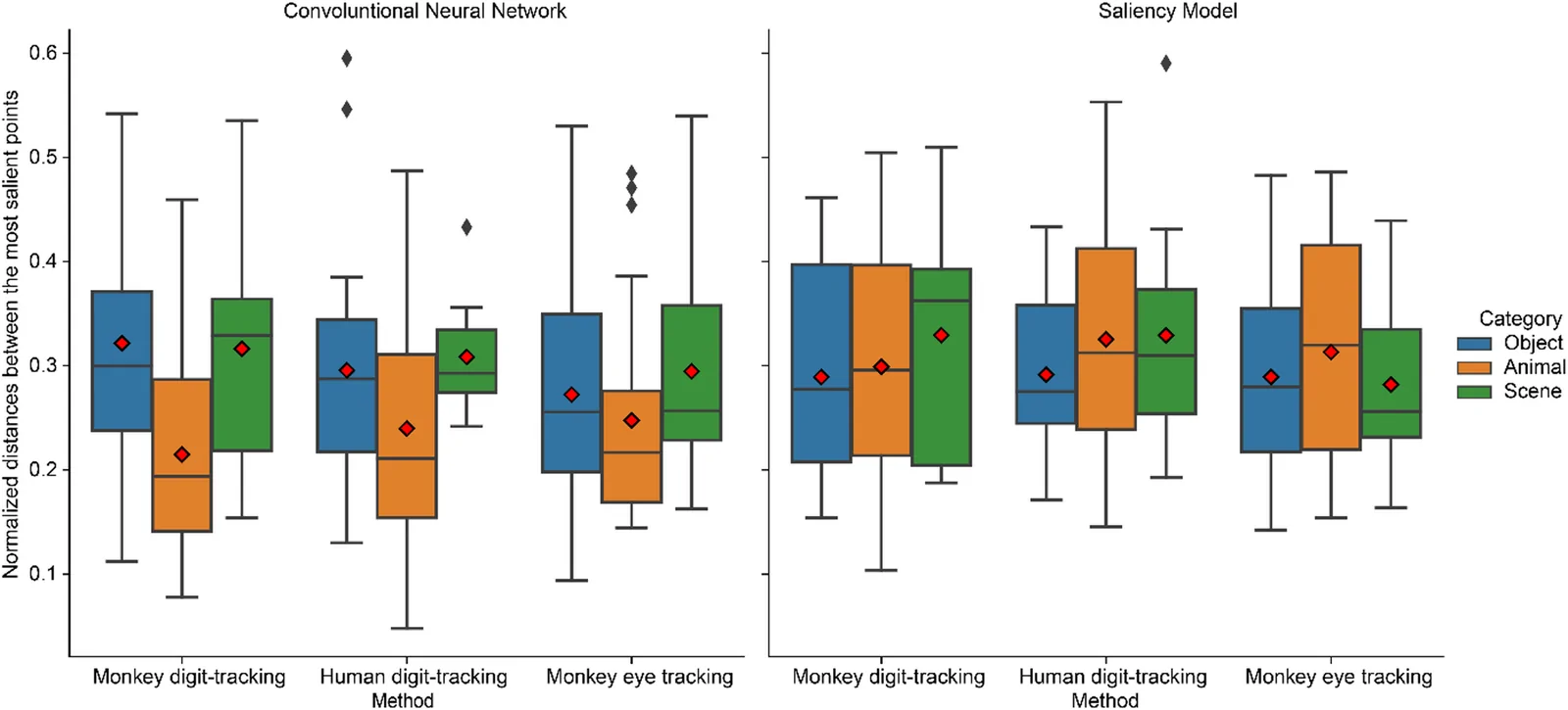
Comparison of spatial alignment between model prediction maps and empirical attention maps. Boxplots show the normalized distance between the most salient points in the model prediction maps and the corresponding empirical attention maps; diamonds indicate mean values. Distances were computed at the image level by comparing model predictions with empirical attention maps averaged across subjects, yielding 59 values per method. For the CNN model, the distance between prediction maps and the attention maps in scene images is slightly shorter even though the test was not significant.

To complement our correlation-based findings, we examined the spatial precision of the models using a distance-based metric. For each image, we identified the most salient point in the model prediction map and in the corresponding empirical attention map, and computed the normalized Euclidean distance between these two locations as an index of spatial alignment. As in the correlation analysis, distances were computed at the image level after averaging empirical attention maps across subjects, yielding 59 distance values per method and model. This alternative metric revealed a marginally significant main advantage for the CNN model overall (*F*(1,58) = 4.381, *p* = 0.041, $\:{\eta\:}_{p}^{2}$ = 0.070). Subsequent separate ANOVAs (Method × Category) showed, for the CNN model, a marginally significant main effect of category (*F*(2,112) = 3.223, *p* = 0.047, $\:{\eta\:}_{p}^{2}$ = 0.103) and a significant interaction effect (*F*(4,112) = 3.219, *p* = 0.015, $\:{\eta\:}_{p}^{2}$ = 0.103), though post hoc tests did not reach significance. As shown in Fig. 6, the CNN’s predicted salient points tended to be closer to empirical data for creature images than for other categories. For the saliency model, no significant effects emerged (method: *F*(2,112) = 0.791, *p* = 0.456, $\:{\eta\:}_{p}^{2}$ = 0.014; category: *F*(2,112) = 0.318, *p* = 0.729, $\:{\eta\:}_{p}^{2}$ = 0.011; interaction: *F*(4,112*)* = 1.759, *p* = 0.142, $\:{\eta\:}_{p}^{2}$ = 0.059).

When combining results from both correlation and distance-based metrics, the CNN model demonstrated superior performance, particularly for human data and socially relevant stimuli. This advantage may reflect its training on human-derived data and capacity to integrate social cues. In contrast, the saliency model showed limited predictive power, with its only robust performance occurring for object images in correlation analyses. Together, these results quantitatively establish that the CNN model better captures attentional patterns across exploration methods, with its performance advantage most pronounced for socially relevant content.

### Shared feature selection in human and macaque visual exploration

**Fig. 7:**
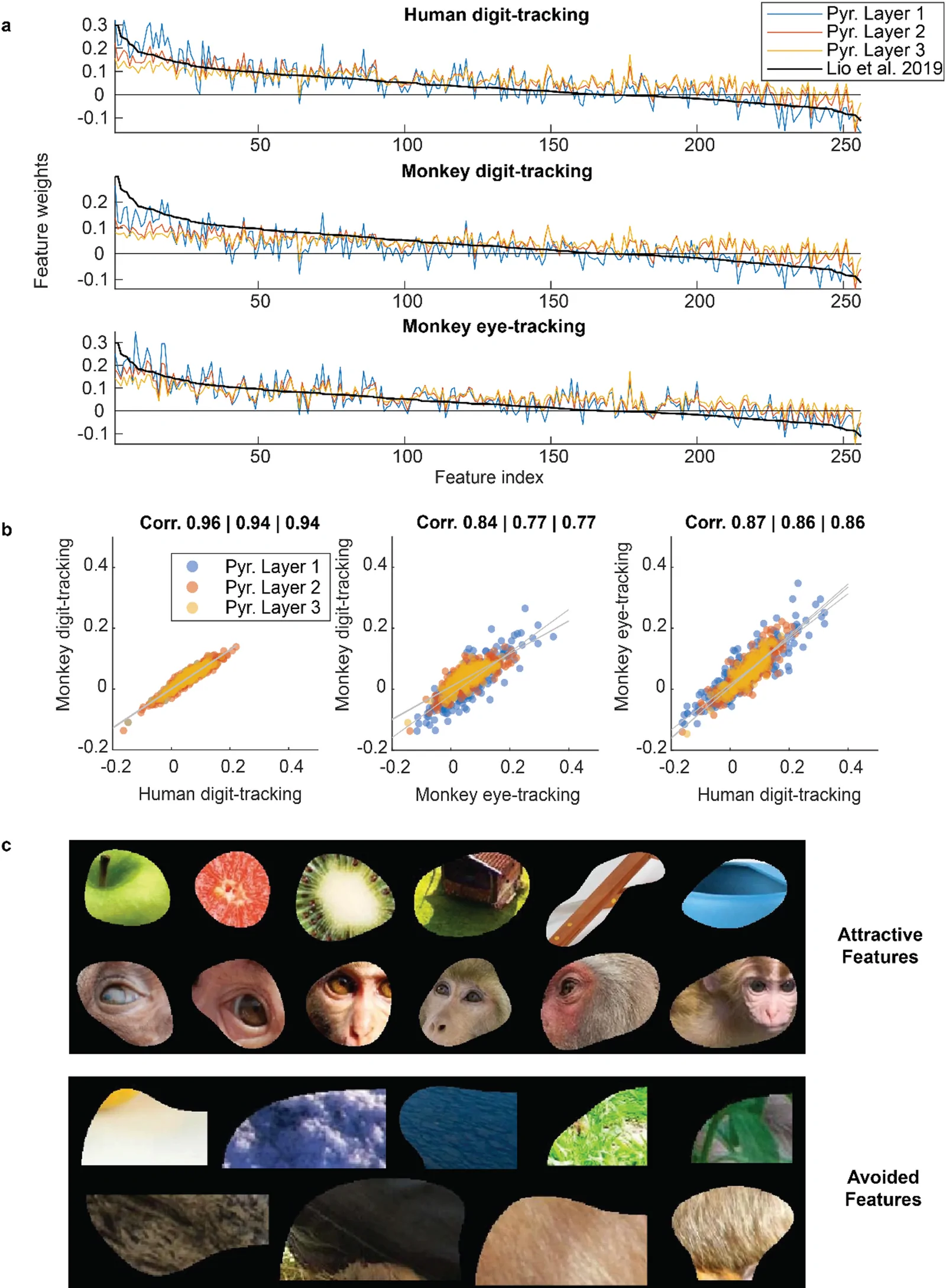
Features decomposed by the Convolutional Neural Network (CNN) architecture. (**a**) Hierarchical orderings of features across testing condition. All conditions closely match the ordering patterns reported by Lio et al.^14^ (black line); (**b**) Scatter plots comparing feature orderings across species and methods. The correlation coefficient between feature orderings of human and monkey digit-tracking was the highest, while the correlations involving monkey eye-tracking were weaker. The correspondence between monkey digit-tracking and eye-tracking showed the greatest variation across pyramid levels. (**c**) Examples of features that were either consistently attractive or avoided across by humans and macaques. Both species were drawn to salient elements such as fruits, objects, eyes, and faces, while they tended to avoid background and peripheral areas.

Building on our previous CNN architecture developed in Lio et al.^14^, we investigated whether humans and macaques attend to similar visual features during exploration. While the previous section’s analyses used the pre-trained model from that study, we extended this approach by retraining the model on our current dataset to compare feature weighting across species and modalities. In our original implementation^14^, we adapted a well-established Convolutional Neural Network (CNN) architecture^18^ to detect essential features during visual exploration. This architecture was initially trained for image classification using the ImageNet database (http://www.image-net.org) for the ImageNet Large-Scale Visual Recognition Challenge^19^. We took the first five convolutional layers and created prediction maps by linearly combining their feature maps. The weights for this combination of feature maps were learned from human empirical data. For the current analysis, we retrained the model on our new dataset to directly compare how features are weighted across different species (humans vs. macaques) and testing methods (digit-tracking vs. eye-tracking). A key aspect of our analysis involved examining how image resolution affects feature detection, through what we refer to as the “pyramid level”, where higher levels correspond to finer image analysis. By manipulating this level, we can examine how the model’s ability to detect and utilize features changes with varying degrees of image detail (Fig. 7).

The results revealed three important findings. First, the relative importance (weighting) of the 256 features in our new analysis closely matched the patterns we previously reported^14^ (Fig. 6a). Second, when we compared how features were weighted between humans and monkeys using digit-tracking, we found remarkably strong correlations at all pyramid levels (0.96, 0.94, and 0.94 for levels 1–3 respectively). Third, the correspondence between monkey eye-tracking and digit-tracking weights became slightly weaker at finer resolutions (0.84 at level 1 to 0.77 at level 3). This pattern suggests that while humans and macaques fundamentally attend to similar visual features during exploration, digit-tracking may capture these features at a slightly coarser resolution compared to eye-tracking, particularly when examining fine details. The high correlations across species indicate shared mechanisms of feature selection, while the resolution-dependent differences highlight how the measurement method can affect the precision of captured attentional patterns.

## Discussion

Digit-tracking, initially designed for human subjects, has proven to be a reliable and innovative tool for studying spontaneous attention allocation in non-human primates in this study. One significant advantage of digit-tracking is its non-invasive nature, which allows data collection in a more relaxed, free-ranging environment, minimizing the stress often associated with traditional laboratory-based methods. We successfully trained five macaques to explore the Gaussian-blurred images on a touch screen within their own cages, achieving stable performance from three of them. Direct comparisons of attention maps revealed that monkey digit-tracking showed significant correspondence with both monkey eye-tracking and human digit-tracking, indicating shared patterns of visual exploration across species and recording modalities. The empirical attention maps further suggested that the explorations were guided by image characteristics and could be predicted by a Convolutional Neural Network (CNN) model. Because the reward schedule was spatially uniform, such spatial biases cannot be attributed to reinforcement contingencies alone. Instead, the visual information itself likely acted as a conditioned reinforcer, drawing exploration toward semantically rich regions. For this reason, we refer to the behavior as curiosity-driven, while acknowledging that it can also be described in purely behavioral terms. The analysis of monkey eye-tracking and human digit-tracking data revealed a similar pattern to monkey digit-tracking, with the most accurate predictions occurring for creature images. The decomposition of the exploration pattern using the CNN model further demonstrated that humans and macaques are attracted to and avoid similar features. On average, 18 sessions were required to reach stable and reproducible performance, which is not particularly difficult to achieve under this experimental setting. This suggests that digit-tracking can provide a reliable method for studying attention allocation in non-human primates.

In this study, digit-tracking data were collected while the subjects were freely behaving in their home cages. The monkeys were not required to remain in front of the screen but instead had to reach a predetermined exploration distance to complete each trial. During training, this exploration distance was gradually increased to the target value. The subjects were generally relaxed and showed no signs of anxiety or resistance to the apparatus, and most adapted to the task rapidly, including individuals with a prior history of being difficult to train in laboratory settings. Despite this overall success, usable data were obtained from only three of the five subjects. Two monkeys developed a strategy of randomly touching the screen to obtain rewards without sustained visual exploration, which limited their inclusion in the final analyses. This reduction in sample size constrains the generalizability of the findings and highlights an inherent challenge of voluntary, self-paced paradigms in nonhuman primates. Ongoing work is aimed at identifying the factors underlying this lack of engagement, including whether it primarily reflects stable individual differences across monkeys or whether participation can be improved through refinements of the experimental design and task structure.

Future studies further address training-related limitations by incorporating online estimates of the proportion of time spent viewing the image during the training phase, allowing early detection of uncooperative strategies and adaptive adjustments to task parameters. In a separate unpublished study, we also explored an alternative trial-termination method in which images were displayed for a fixed duration triggered by the first contact, after which the subject received the reward regardless of the exploration distance achieved. Although this approach was not adopted in the present study due to concerns about whether it would elicit sufficient exploration behavior, preliminary observations indicated that subjects continued to interact with the screen even under the fixed-duration condition. This suggests that their interactions were driven, at least in part, by intrinsic motivation to acquire visual information, and this paradigm may represent a promising alternative for future experiments.

The use of identical stimuli throughout the training phase represents a further limitation of the present study. Because task performance stabilized while the same images were repeatedly presented, exploratory behavior during later stages may have been influenced in part by stimulus familiarity, and not just online visual attention. In subsequent and ongoing studies using this paradigm, this issue has been addressed by introducing distinct image sets for the training and testing phases, allowing test performance to be evaluated with novel stimuli and providing a clearer assessment of visually driven exploratory behavior.

The stimulus set in the present study was designed to encompass a wide range of visual content in order to capture naturalistic exploratory behavior, rather than to tightly control specific stimulus dimensions. Within this framework, images were grouped into broad categories reflecting differences in attentional relevance and semantic significance, rather than to serve as strictly matched experimental conditions. Future studies can build on this approach by using more systematically controlled stimuli to isolate specific factors of interest. For example, in the unpublished study mentioned above, face stimuli were used, and variables such as species, gender, and familiarity were explicitly balanced and controlled. Such designs will allow a more precise examination of how specific image properties shape exploratory behavior while retaining the advantages of the digit-tracking paradigm.

Despite the limitations of our experimental approach, attention allocation analyses revealed striking cross-species and cross-method similarities. Direct comparisons of empirical attention maps showed that monkey digit-tracking maps were significantly correlated with both monkey eye-tracking and human digit-tracking maps, indicating robust common structure across recording methods. The CNN model’s consistent superiority over the saliency model – particularly for social stimuli (faces, eyes, and bodies of conspecifics) confirms that macaque attention, like human attention, prioritizes meaningful features beyond raw visual salience^20–22^. Although the CNN showed slightly reduced accuracy for monkey digit-tracking versus eye-tracking, the preserved hierarchical orderings of features across all conditions suggest this primarily reflects measurement precision differences rather than distinct attentional strategies. Most importantly, the conserved feature importance patterns imply an evolutionarily shared attention mechanism that operates similarly whether exploration occurs via fingers or eyes.

In conclusion, our findings demonstrate that digit-tracking can be a valuable, adaptable tool for studying spontaneous visual behavior in non-human primates. Monkeys explored information-rich regions of the image display, in a manner consistent with established metrics of visual attention. This research contributes to a broader understanding of attention mechanisms in primates, showing that visual exploration involves an intrinsic component related to curiosity. Our findings open up new avenues for research on the cognitive and motivational foundations of attention, as well as practical applications for studying primate behavior outside the constraints of traditional lab environments. By demonstrating a method that could ultimately benefit a range of animal research, from controlled lab studies to free-ranging wildlife observation, this study introduces digit-tracking as a flexible, scalable, and insightful approach for primate visual cognition research.

## Methods

### Participants

For this study, we collected data from five macaques at ISC-MJ, including two rhesus macaques (Monkey V, male, 11.2 kg, 11 years old; Monkey Z, female, 8.8 kg, 19 years old) and three long-tailed macaques (Monkey T, male, 9.5 kg, 12 years old; Monkey S, male, 9.7 kg, 12 years old; Monkey J, male, 9.9 kg, 12 years old). In addition, we obtained the eye-tracking data from Monkey V and Monkey T. All procedures conducted on the animals were approved by the national ethics committee on animal experimentation N°42 (CELYNE) and by the French Minister of Research and Innovation, conformed to European and National Institutes of Health Guidelines for the Care and Use of Laboratory Animals and complied with ARRIVE guidelines.

Animals were provided with a diet consisting of monkey chow, fresh fruits, and vegetables, and their water intake was regulated. Each morning, the monkeys received a minimum of 50 mL of water with their daily portion of monkey chow, and the task began shortly after the water and food were provided. During the task, they received approximately 0.5 mL of water for each successfully completed trial. The total daily water allowance was adjusted based on each monkey’s performance: if a monkey did not complete the entire set of images in a single session, the water provided after the task was gradually reduced until they consistently completed the full set in one session, after which the acquisition phase was initiated. Throughout the study, the minimum daily water intake for each animal was maintained at no less than 20 mL per kilogram of body weight, regardless of task performance, to ensure proper hydration and overall health. Finally, each monkey had one day of free access to water each week. Enrichments were offered several times a week with different toys or substrates (boxes and puzzles containing dry fruit, swings) that promote play, curiosity, object manipulation, and foraging, following recommendations from our own laboratory animal welfare committee.

A group of nine human participants (mean age = 27, SD = 5.7) were recruited from the pool of graduate students and technicians of the Institute of Cognitive Science Marc Jeannerod of Bron (ISC-MJ), France (FR). All participants gave written informed consent prior to participating, reported normal or corrected-to-normal vision, and reported no history of neurological or psychiatric disorders. All the experiments followed all guidelines and recommendations and were approved by the local Ethics Committee in accordance with the Declaration of Helsinki.

### Picture database

A total of 59 images depicting various aspects of natural and social scenes, including humans, monkeys, other animals, landscapes, objects, and abstract art, were selected. The selection of images for the database intentionally encompassed a wide range of content to ensure that it did not limit the exploratory behavior and maintained the curiosity of both the tested human and monkey subjects. The picture database includes images featuring the bodies and faces of both humans and monkeys because these stimuli elicit distinctive exploration patterns that serve as an ideal basis for conducting comparative analyses across different species and contexts. Images were categorized into three groups: object (20), scene (12), and creature (27).

### Digit-tracking method

The digit-tracking method was implemented following the same framework described in Lio et al.^14^. Stimulus presentation, real-time picture modification, and data recording were written using the Psychophysics Toolbox for MATLAB (R2015a, the MathWorks, Inc.).

This method consists of presenting, on a capacitive touchscreen display, a blurred image simulating an approximation of the acuity present in peripheral vision. When the subject touched the screen with its finger, an area approximating the size of the fovea (see details below) was displayed at full resolution just above the point of contact. The area was shifted upwards to prevent the subject’s view from being masked by the finger. Removing the finger from the display restored the image’s fully blurred appearance and sliding the finger across the display caused the full resolution area to be updated. Coordinates of the area’s center, regarded as the proxy for eye positions and attention, were continuously recorded.

In the implementation of the digit tracking method, three critical parameters need to be accounted for: the degree of picture degradation to simulate an approximation of peripheral vision, the dimensions of the foveal area being simulated, and the distance of the foveal aperture relative to the point of contact. We applied a Gaussian blur filter with a size parameter of σ = 1.35° to simulate a more pronounced blur compared to standard peripheral vision as it has previously demonstrated that slightly higher blur level promotes image exploration^15^. Additionally, given that the monkeys tested in their home cage with the digit-tracking method were not head-fixed, the distance between their eyes and the screen could not be controlled precisely. However, this distance could not be less than 16.5 cm, as it corresponded to the distance between the screen and the bars of the home cage. Therefore, applying a more intense blur guaranteed that the image resolution remained below the acuity of peripheral vision, even under the most minimal eyes-screen distance of 16.5 cm. A Gaussian aperture window with a size parameter of σ = 2.6° was used to simulated foveal vision. The aperture window size was slightly enlarged compared to that used in the original study^14^ to encourage exploration in monkeys. The distance between the point of contact and the center of the foveal vision was 1.4°.

### Eye tracking method

As subjects V and T were taking part in electrophysiology experiments of other studies, these individuals were equipped with a head-restraint device that facilitated the recording of their eye positions using an infrared eye tracker (ISCAN ETL200 Series, sampling rate = 240 Hz) while they explored the images. The open source software NIMH MonkeyLogic was used for task control and data acquisition^23^. Each session began with a calibration routine of 9 points using a fixation task. Throughout the experiment, the stability of the calibration was continuously monitored by the operator, and if any deviation or inconsistency was detected, the calibration process was repeated to ensure accurate and reliable eye-tracking data.

### Apparatus and procedures

The experiment consisted of recording the exploration of pictures by both monkeys and humans. Monkeys’ explorations were tracked using either the digit-tracking method in their home cage or eye-tracking in the laboratory. For humans, only the digit-tracking method was used.

The task structure remained consistent across all conditions, but participants interacted with the screen differently depending on whether they were tested using the digit-tracking or eye-tracking methods. In the digit-tracking condition, each trial began with a green virtual button (7.1°) appearing at the center of the screen against a black background. The subject initiated the presentation of the blurred image by touching this button. The subject could then freely explore the image until a track-path of a predetermined length (2250 pixels at a screen resolution of 188 pixels per inch, corresponding to ≈ 61.5° of visual angle) had been reached. The image was then removed and monkey subjects were rewarded with a drop of juice. After a fixed 2-second delay to allow the reward to be consumed, the next trial began. Trials continued until all images of the database had been explored, with the order of their presentation following a pseudo-random sequence.

All monkeys underwent identical familiarization and training procedures as follows: in the beginning, subjects were only required to touch the green virtual button to receive a reward until they became accustomed to interacting with the touchscreen, typically within fewer than 2 sessions. Subsequently, blurred images were introduced upon button touch, and the track-path to reach trial completion was gradually extended. The required exploration length started at 50 pixels and was increased in 50-pixel increments on each new trial until the final criterion of 2250 pixels was reached. Monkeys typically required only 2–3 sessions to perform consistently at this level. The choice of the 2250-pixel path length was informed by pilot studies in other monkeys, where this value was found to balance two competing constraints: (i) it allowed us to present relatively large image sets (50–60 images) within a single session without animals disengaging from the task, and (ii) it ensured that a sufficient amount of exploration data could be collected across repeated presentations of the same images. Longer distances were not tested systematically, as our main strategy to maximize exploration data was to repeat the image set across multiple sessions and days, thereby minimizing habituation and allowing us to average across repetitions to overcome the noise of single-trial exploration maps.

To test subjects in their home cage with the digit-tracking method, a tablet computer (Dell Latitude 7210, frequency of 60 Hz, resolution of 1920 × 1280 pixels, 57.5° × 46.3° of visual angle) was integrated into a mobile, battery powered training box equipped with a liquid delivery system and a seating platform. This mobile device was positioned against the bars of the subject’s cage, providing access to the touchscreen to perform the task.

In the eye-tracking condition, monkeys were seated in a primate chair facing a capacitive touchscreen (ELO^®^ 2770 L, frequency of 60 Hz, resolution 1920 × 1280 pixels, 55.9° × 44.9° of visual angle). Their heads were immobilized to enable eye-tracking, as described above. The dimensions of the images were adjusted to ensure that their sizes in visual angles were similar to those used in the macaque digit-tracking condition. The structure of the task was similar to that of the digit-tracking condition, except that image presentation was initiated by fixation of a green target for 500 milliseconds. To ensure comparable exploration in both the macaque digit-tracking and eye-tracking experiments, the images were displayed for 2 s. This duration corresponded to the average time necessary to achieve 56° of visual angle exploration, which was the cumulative distance needed to complete a trial in the digit-tracking experiment. Subjects were required to keep their eye position within the screen limits during the trials. Exceeding these limits terminated the trial without reward delivery, and the image was presented again later in the session. Subjects underwent training until they achieved a performance level of 80% successful trials before beginning formal data acquisition.

In the human digit-tracking condition, participants were instructed to sit in front of the same tablet computer used in the macaque digit-tracking condition (Dell Latitude 7210, frequency of 60 Hz, resolution 1920 × 1280 pixels, 57.5° × 46.3° of visual angle) without any constraint. Each participant was instructed to freely explore each picture during a single session lasting approximately 30 min. The picture was removed when the cumulative distance of exploration reached 4000 pixels (≈ 73.0° of visual angle). The selection of this distance was guided by participant feedback collected in a preliminary pilot study to enable sufficient image exploration without inducing disinterest and to maintain participants’ engagement throughout the task.

### Data analyses

All the analyses were conducted in MATLAB (R2015a, the MathWorks, Inc.) using our custom scripts. Some plots were generated in Python (3.9.19) using the Seaborn library (0.13.12). Statistical tests were conducted in JASP^24,25^.

### Data inclusion

Task engagement during digit-tracking was quantified using a temporal engagement metric defined as the proportion of total exploration time spent within the image boundaries. This metric was used to assess whether subjects were actively interacting with the visual content of the image, rather than touching areas outside the image region to obtain rewards. For each trial, the engagement metric was computed as the cumulative duration during which the finger position remained within the image area, divided by the total exploration duration. Trials with an engagement proportion below a predefined threshold were excluded from further analyses. Based on empirical inspection and to ensure that attention maps reflected image-guided exploration, this threshold was set to 0.8 (i.e., at least 80% of exploration time within the image boundaries). After applying this criterion, participants for whom the remaining number of valid sessions was insufficient to support reliable attention map estimation were excluded entirely from subsequent analyses.

### Attention maps

Attention heat maps were computed as described in Lio et al.^14^ and Yang et al.^15^. Briefly, tracking point coordinates were converted from screen space to picture space. The attention map for each subject and each picture, defined as the exploration probability density, was calculated from the tracking data using kernel density estimation with a Gaussian kernel weighted by duration and a bandwidth of 1° of visual angle. Each subject-level density was then normalized using a Min-Max scaling process. Finally, group-level maps were generated by averaging scaled subject-level densities, after which they were normalized using the same normalization procedure. Attention maps for eye-tracking data were computed in an analogous way, based on segmented fixations.

### Convergence analysis

For each image and each iteration, we generated a random permutation of session numbers of this image. Then we averaged attention maps from 1 to N-1 sessions (N = number of sessions for each image) and computed the correlation between these attention maps and the average attention map across all sessions. This procedure was repeated 40 times and then we plotted the curves of the mean coefficient of determination over sessions. We set the criterion for explanation variance at 0.9 and computed the number of sessions needed to reach this criterion.

### The saliency maps

The saliency maps were generated using the approach described by Walther and Koch^17^, which is a variation of the classical saliency model originally proposed by Itti and Koch^26^. The authors developed a Saliency Toolbox written in MATLAB and made it available on GitHub (https://github.com/DirkBWalther/SaliencyToolbox). We used this toolbox with its default parameters to produce the saliency maps.

### The convolutional neural network model

The Convolutional Neural Network model was trained by Lio et al.^14^. We utilized the pre-trained AlexNet Network^18^ to generate prediction maps. The selected model was trained on a subset of more than one million images, extracted from the ImageNet database (http://www.image-net.org) for the ImageNet Large-Scale Visual Recognition Challenge (ILSVRC^19^. Dedicated originally for large-scale image classification, the CNN architecture was transformed using the Matlab Neural Network toolbox (Mathwork Inc.) to solve a regression problem and generate predictions. First, the outputs of the fifth convolutional layer, rectified by a linear unit (ReLU layer), were interpolated to obtain 256 feature maps with the same resolution as the input image (227 × 227 pixels). Then the maps were linearly combined using wCorr, a fast estimate of the weights by averaging the Pearson Correlation Coefficients calculated between a feature map and the measured saliency map (the 256 weights are learned independently). This approach can be considered as a simplified implementation of the Kümmerer et al.^27^ method for saliency map prediction.

### The distances between most salient points

First, we identified the most salient point (i.e., the pixel with the highest value) in each map. For each image, we had one prediction map from the saliency model and one prediction map from the CNN model, but multiple empirical attention maps across different subjects or sessions. We then measured the distance between the most salient point in each model’s prediction map and the most salient point in each empirical attention map, normalized by the diagonal length of the image. Finally, we averaged these distances for each image within each recording method.

## Supplementary Information

Below is the link to the electronic supplementary material.


Supplementary Material 1


## Data Availability

The example code and data are available on the Open Science Framework (https://osf.io/na4mv/).
