# Supplementary material for: Digit-tracking reveals curiosity-driven visual attention in macaque monkeys
Source: Sci Rep. 2026 Jun 14;16:27117. doi: 10.1038/s41598-026-57654-4 (PMC13527078; doi:10.1038/s41598-026-57654-4)
Supplement: Supplementary file 1 [file 41598_2026_57654_MOESM1_ESM.docx]

Figure S1. **Examples of blurred images placed on the black screen.** The three image show examples from scene, object, and animal categories, respectively. The relative ratio of image and screen and the blur level replicate the same settings of the experiment.

Figure S2. **Representative examples of finger movements from one human participant and two monkey participants using digit-tracking interface.** We chose the image and randomly took one trial from them. The image was placed at the center of the black screen, reflecting the real setting of the experiment. The white half-transparent points represent the tracking points and the red lines represent the scan paths. All participants adopted a smooth and continuous scanning strategy during exploration.

Figure S3. **Subject-level comparisons between model prediction maps and monkey digit-tracking attention maps.** The left panel shows the correlation coefficients between monkey digit-tracking attention maps and Convolutional Neural Network prediction maps. The mean correlation coefficients for each subject across image categories were as follows: Monkey T, 0.237 (SD = 0.290) for object, 0.358 (SD = 0.175) for animal, 0.059 (SD = 0.145) for scene; Monkey V, 0.275 (SD = 0.262) for object, 0.373 (SD = 0.242) for animal, 0.110 (SD = 0.166) for scene; Monkey Z, 0.195 (SD = 0.233) for object, 0.345 (SD = 0.246) for animal, 0.006 (SD = 0.120) for scene. The right panel shows the correlation coefficients between monkey digit-tracking attention maps and the saliency model prediction maps. The mean correlation coefficients for each subject across image categories were as follows: Monkey T, 0.137 (SD = 0.141) for object, 0.085 (SD = 0.113) for animal, 0.011 (SD = 0.080) for scene; Monkey V, 0.209 (SD = 0.156) for object, 0.079 (SD = 0.109) for animal, 0.068(SD = 0.120) for scene; Monkey Z, 0.110 (SD = 0.086) for object, 0.045 (SD = 0.077) for animal, -0.040 (SD = 0.075) for scene. Consistent with the group-level effects, subject-level analyses (separate Model × Category ANOVAs for each animal) confirmed that CNN models reliably outperformed Saliency models across all individuals (all ps < 0.001). Furthermore, the superior performance of CNN in animal images were consistently observed in each subject, indicating that these effects are robust at the individual level. *p < 0.05, **p < 0.01, ***p < 0.001.
